# Proposing an explanatory framework based on the fear-avoidance model: a mixed-methods analysis of kinesiophobia in patients after percutaneous coronary intervention in home-based cardiac rehabilitation

**DOI:** 10.3389/fcvm.2026.1819126

**Published:** 2026-06-04

**Authors:** Li Sun, Chunlei Nie, Dandan Xu, Lan Wei, Weiwei Zong, Yanli Hu, Yuan Li, Xinyue Zhang

**Affiliations:** 1 Department of Cardiology, The First Affiliated Hospital with Nanjing Medical University, Nanjing, China; 2 School of Humanities, Southeast University, Nanjing, China; 3 Department of Health Management Center, Nanjing Drum Tower Hospital, Affiliated Hospital of Medical School, Nanjing University, Nanjing, China; 4 School of Nursing, Nanjing Medical University, Nanjing, China

**Keywords:** coronary artery disease, fear-avoidance model, home-based rehabilitation, kinesiophobia, mixed-method

## Abstract

**Introduction:**

Exercise-based cardiac rehabilitation is safe and effective for patients with coronary artery disease, with home-based programs typically delivered through digital platforms (e.g., mobile applications, wearable devices) that enable remote monitoring and personalized guidance. However, home-based rehabilitation, while offering flexibility in time and location, is frequently impeded by kinesiophobia. This study aimed to identify the determinants of kinesiophobia in patients following percutaneous coronary intervention and to examine their interplay in driving maladaptive fear and influencing engagement in rehabilitation.

**Methods:**

This study adopted a sequential explanatory mixed-methods design. A cross-sectional study was conducted to investigate kinesiophobia among hospitalized patients, followed by semi-structured interviews with patients exhibiting high levels of kinesiophobia selected through purposive sampling from the cross-sectional cohort.

**Results:**

The quantitative study involved a cross-sectional survey of 220 post-PCI patients, with a mean age of 57.85 ± 12.17 years. The majority were male (*n* = 170, 77.3%), living with a partner (*n* = 192, 87.3%), and had comorbid chronic conditions (*n* = 153, 69.5%). Multiple linear regression analysis revealed that living alone (*β* = 0.17, 95% CI: 1.53–6.45), urban residence (*β* = 0.16, 95% CI: 0.16–5.05), previous PCI history (*β* = 0.11, 95% CI: 0.15–4.21), comorbidities (*β* = 0.13, 95% CI: 0.41–3.91), clinical symptom severity (*β* = 0.23, 95% CI: 0.23–0.61), and lower cardiac rehabilitation knowledge scores (*β* = −0.43, 95% CI: −0.24–0.15) significantly predicted kinesiophobia levels, collectively explaining 46.5% of the variance in Tampa Scale for Kinesiophobia (TSK) scores. Building upon these quantitative findings, 18 participants (7 females, 11 males) with high kinesiophobia scores (TSK > 37) were purposively sampled for the qualitative phase. Fourteen open codes were collapsed into eight focused codes, which subsequently resulted in the following four themes: 1) catastrophic interpretation of somatic Sensations, 2) knowledge deficits and misinformation sustaining Fear, 3) vicious cycle of symptom misinterpretation and knowledge Gaps, and 4) Positive perception.

**Conclusion:**

Based on integrated findings, this study proposes an explanatory framework derived from the Fear-Avoidance Model, delineating three core pathways: the initial origination of fear through symptom misinterpretation, its subsequent amplification via cognitive and environmental factors, and its eventual consolidation through behavioral reinforcement via avoidance cycles.

## Introduction

Cardiovascular disease remains a leading cause of global mortality, with coronary artery disease (CAD) representing a substantial burden (1). Percutaneous coronary intervention (PCI) serves as a primary revascularization strategy for CAD, significantly improving symptoms and prognosis (2). However, without appropriate rehabilitation management, patients remain vulnerable to recurrent cardiovascular events (3). Exercise-based cardiac rehabilitation (EBCR), classified as a Class ⅠA intervention in both domestic and international guidelines, significantly decreases cardiovascular mortality by 42%, hospital readmissions by 42%, and the risk of recurrent myocardial infarction by 28% (4). Despite established efficacy, traditional center-based EBCR faces implementation challenges including geographical barriers, transportation difficulties, and substantial costs, particularly for patients in remote areas or with limited mobility (5). Additionally, while center-based programs offer important opportunities for social cohesion and peer support among participants, these benefits remain inaccessible to many patients due to various realistic constraints (6, 7). Home-based cardiac rehabilitation has emerged as an effective alternative that can overcome these accessibility barriers while maintaining clinical benefits (8, 9). Nevertheless, new challenges have emerged in this home-based context, with psychological barriers appearing particularly prominent (10). Furthermore, fear of exercise can negatively impact adherence to home-based cardiac rehabilitation programs (11).

Kinesiophobia, which is an irrational fear of movement and exercise, serves as a significant psychological barrier (12, 13). While a moderate level of fear can serve a protective role, excessive fear of non-threatening activities can be detrimental. Research indicates that approximately 75.7% of CAD patients experience varying degrees of exercise-related fear, which correlates negatively with recovery outcomes (14). Elevated levels of kinesiophobia in CAD patients adversely affect rehabilitation results, leading to reduced compliance with exercise regimens and declines in both aerobic capacity and muscle strength, ultimately increasing the risk of cardiac events and mortality (15), 20% of participants reported a high level of kinesiophobia at the onset of remote EBCR, which decreased to 10% at follow-up. This finding aligns with a study by Hoeve et al. (16), which found that 40% of a mixed population of patients with heart disease exhibited high levels of kinesiophobia at the start of center-based cardiac rehabilitation, decreasing to 26% post-rehabilitation. While it is reasonable to hypothesize that a greater proportion of patients with kinesiophobia may prefer center-based EBCR, it is encouraging that participation in remote EBCR also contributes to a reduction in kinesiophobia. Previous research has extensively examined the specific causes and psychological impacts of kinesiophobia among patients who have undergone PCI (17, 18). Key factors influencing kinesiophobia have been identified, with education level, monthly income, anxiety, and exercise self-efficacy consistently associated with its occurrence. The prevalence of kinesiophobia among patients with heart disease is notably high and influenced by multiple determinants (19). The home-based rehabilitation environment is multifaceted, and the scope and contributing factors of kinesiophobia are not yet fully understood. Thus, there is a pressing need to investigate kinesiophobia among CAD patients within a home setting.

This study is grounded in the Fear-Avoidance model and employs an explanatory sequential mixed-methods design. The quantitative phase first investigates the current status and influencing factors of kinesiophobia among post-PCI patients in home-based rehabilitation settings, followed by a qualitative phase that enriches and contextualizes the quantitative findings through in-depth interviews. By examining both the quantitative relationships between kinesiophobia and rehabilitation engagement and qualitatively exploring patients' lived experiences, this study aims to develop a comprehensive understanding of this complex phenomenon and identify potential targets for intervention to improve rehabilitation adherence and outcomes.

## Materials and methods

### Study design

This study employs an explanatory sequential mixed-methods design. Initially, quantitative data were collected and analyzed, followed by qualitative data collection to further explore the quantitative findings. The findings from this inductive analysis were subsequently integrated with quantitative results to inform the development of an explanatory framework based on the Fear-Avoidance Model. Each participant signed an informed consent form. The study protocol was approved by the Hospital Ethics Committee (Approval No. 2023-SR-227). All methods adhered to relevant guidelines outlined in the Helsinki Declaration.

### Participants

During hospitalization, each patient received a supervised, individualized exercise regimen (e.g., limb exercises, a modified version of the Eighth Set of Radio Calisthenics). Upon discharge, this was transitioned to a tailored home-based exercise rehabilitation program. Subsequent exercise prescriptions were then regularly delivered through a WeChat-based follow-up system. Participants were selected through purposive sampling from CAD patients discharged from Jiangsu Province Hospital between October 2024 and July 2025. Inclusion criteria included: (1) diagnosed with coronary heart disease and undergoing post-PCI; (2) ability to speak Chinese and communicate effectively; (3) capacity to understand and cooperate during the interview; and (4) participated voluntarily in a hybrid exercise rehabilitation program, consisting of a 2–3 day in-hospital phase followed by a one-month home-based phase. Exclusion criteria comprised: (1) heart, brain, or kidney conditions preventing exercise rehabilitation; (2) severe anxiety, depression, or other mental health issues inhibiting cooperation; and (3) lack of informed consent. According to the sample size criteria for linear regression proposed by Riley et al. (20), Based on an anticipated adjusted R^2^ of 0.20 (informed by previous studies) and 13 candidate predictor parameters, a minimum of 209–259 valid responses was required for this study. Accounting for a 10% invalid response rate, we aimed to recruit 232–288 participants.

For the qualitative component, participants were initially purposively sampled from those who scored above 37 on the kinesiophobia scale to capture individuals with clinically significant fear (21). Considering that patients with high levels of kinesiophobia (all with TSK scores >37) may include both relatively lower and higher scores, and that their cognitive and emotional experiences may differ substantially, maximum variation sampling was subsequently applied to ensure diversity in kinesiophobia levels within this group.

### Data collection

#### Quantitative study

Data collection occurred one-month post-discharge through telephone interviews, online platforms, or outpatient follow-ups. General information for the questionnaire was gathered by researchers via electronic medical record reviews and direct inquiries. Patients' baseline characteristics included: (1) sociodemographic factors such as sex, age, education, income, residence, work, PCI history, times of hospital admission, and other comorbid chronic disease; and (2) clinical assessment tools selected based on the core dimensions of the Fear-Avoidance Model (22), including the Tampa Scale for Kinesiophobia (TSK), clinical symptom scores, the Cardiac Rehabilitation Inventory (CRI), and the General Self-Efficacy Scale (GSES).

Clinical Symptoms and signs were evaluated using a symptom scoring method (23, 24), which assesses the frequency and severity of typical cardiac symptoms, including chest pain, palpitations, dyspnea, and fatigue. Higher scores indicate more pronounced clinical symptoms.

The Tampa Scale for Kinesiophobia (25) is a 17-item self-report questionnaire assessing fear of movement and (re)injury. Items are scored on a 4-point Likert scale (1 = strongly disagree to 4 = strongly agree), with total scores ranging from 17 to 68. Higher scores indicate greater kinesiophobia. The Chinese version has demonstrated good reliability (Cronbach's *α* = 0.859).

Cardiac Rehabilitation Inventory (26) is an 18-item scale that assesses patients' understanding of rehabilitation components, including outcome anxiety, process anxiety, and autonomy. Items are rated on a 5-point Likert scale, with higher scores indicating a greater need for individualized cardiac rehabilitation support. The Chinese version has demonstrated good reliability (Cronbach's *α* = 0.816).

The General Self-Efficacy Scale (27) was used to measure individuals' overall self-efficacy. The scale consists of 10 items rated on a 4-point Likert scale from 1 (not at all true) to 4 (exactly true), with total scores ranging from 10 to 40. Higher scores reflect greater self-efficacy. Across studies in different countries, the GSES has shown Cronbach's *α* coefficients ranging from 0.75 to 0.94 and test-retest reliability from 0.55 to 0.75 (27).

#### Qualitative study

For the qualitative component, following completion of the initial quantitative assessments, respondents who scored above 37 on the TSK were contacted by telephone. The interview guide was developed based on the research objectives, quantitative findings, and team discussions, and was then pilot-tested with two eligible patients who were not included in the final sample. Before the interviews began, the project leader (author XYZ, master's degree), who was also the head nurse of the ward during the patients' hospitalization, explained the purpose and procedures of the study to the participants and introduced the interviewer. The interviews were conducted by a female clinical nurse (author YL) holding a master's degree in nursing and trained in qualitative research methods, who had no pre-existing relationship with the participants, as they were from different centers and had no prior clinical contact. After receiving comprehensive information, participants voluntarily signed informed consent forms.

Each interview then commenced with a friendly conversation and an open-ended question. “Can you tell me your thoughts or feelings during the CR program?” After this open question, subsequent questions were asked to understand the reason for their Kinesiophobia. Some probing questions were asked, such as “why do you feel anxious, scared, or uncomfortable while exercising?” or “can you tell me a little bit more about what are you scared about?” The interviews were flexible enough to generate richer information. All interviews were audio-recorded, and field notes were taken to capture non-verbal cues and contextual details. To document personal assumptions and potential biases, the interviewer maintained a reflexive journal throughout the data collection and analysis process. Regular debriefing sessions were held with a senior researcher (author CLN, doctoral degree) experienced in mixed-methods research to critically examine emerging interpretations and minimize the influence of preconceptions. Data collection continued until theoretical saturation was reached, defined as the point at which three consecutive interviews yielded no new codes or themes, with each interview lasting approximately 20–30 min.

### Data analysis

Statistical analyses were conducted using SPSS version 25.0. Categorical data were summarized using frequencies and percentages. Continuous data that were normally or approximately normally distributed were reported as means and standard deviations. Group comparisons were performed using independent-samples *t*-tests or one-way analysis of variance (ANOVA), as appropriate. A multiple linear regression analysis was conducted with the total kinesiophobia score as the dependent variable, incorporating independent variables that exhibited statistically significant differences in the univariate analyses. A *p*-value of less than 0.05 was deemed statistically significant.

For the qualitative data, Colaizzi's seven-step phenomenological analysis method was used to inductively explore participants' lived experiences. NVivo (version 11) facilitated data management and coding. Within 24 h post-interview, two researchers (LS & DDX) independently transcribed the audio recordings verbatim and coded the transcripts line by line. Initial codes were generated inductively. The coders compared coding structures and resolved discrepancies through discussion. Related codes were grouped into subthemes and then aggregated into overarching themes, with the coding framework iteratively refined throughout. Results from quantitative and qualitative analyses were integrated using a joint display (28) to construct a proposed explanatory framework based on the Fear-Avoidance Model.

## Results

### Quantitative findings

A total of 231 questionnaires were distributed, with 220 valid responses received, resulting in an effective response rate of 95.2%, and the 220 participants had a mean age of 57.85 ± 12.17 years. Most participants were male (*n* = 170, 77.3%). 87.3% lived together with a partner (*n* = 192); 69.5% had comorbid chronic conditions (*n* = 153); 20% had a history of PCI (*n* = 44). Other sociodemographic and clinical characteristics of the participants are shown in Table 1. After Spearman correlation analysis, it was found that TSK scores were correlated with cardiac rehabilitation scores (*F* = −0.536, *P* < 0.01) and clinical symptom scores (*F* = 0.429, *P* < 0.01), but no significant correlation was observed with self-efficacy scores (*F* = −0.122, *P* = 0.071).

**Table 1:** Characteristic of participants.

Factors	Total (*N* = 220), *n* (%)	Point of TSK (mean ± SD)	*t/F*	*p* value
Gender			2.633	0.010
Male	170 (77.3%)	51.69 ± 7.59		
Femal	50 (22.7%)	55.08 ± 8.13		
Age			−3.071	0.002
60 or younger	113 (51.4%)	50.91 ± 7.81		
61 or older	107 (48.6%)	54.09 ± 7.54		
Residential Status			−2.515	0.013
Living together	192 (87.3%)	51.96 ± 7.71		
Living alone	28 (12.7%)	55.89 ± 7.86		
Residence			−2.857	0.005
City	129 (58.6%)	53.71 ± 6.90		
Town	91 (41.4%)	50.69 ± 8.72		
Education			5.418	0.005
college degree and above	53 (24.1%)	55.00 ± 7.21		
Senior high schoolor technical secondary school	111 (50.1%)	52.41 ± 7.24		
Junior high school and below	56 (25.4%)	50.16 ± 8.85		
Monthly income (RMB)			5.497	0.005
>8,000	44 (20.0%)	55.43 ± 7.56		
5,000–8,000	100 (45.5%)	52.55 ± 7.40		
<5,000	76 (34.5%)	50.62 ± 8.08		
Work status			−1.478	0.141
Working	143 (65.0%)	51.89 ± 8.03		
None or Retired	77 (35.0%)	53.52 ± 7.37		
PCI history			−4.242	0.000
Yes	44 (20.0%)	56.77 ± 8.07		
No	176 (80.0%)	51.38 ± 7.41		
Times of hospital admission			1.166	0.314
0	127 (57.7%)	52.65 ± 7.95		
1–2	69 (31.4%)	51.49 ± 6.76		
Over 3	24 (10.9%)	54.21 ± 9.80		
Other comorbid chronic disease			−4.083	0.000
Yes	153 (69.5%)	53.84 ± 8.04		
No	67 (30.5%)	49.31 ± 6.33		

Linear regression results in Table 2 showed that patients with living alone (*β* = 0.17, 95% CI: 1.53–6.45), living in city (*β* = 0.16, 95% CI: 0.16–5.05), PCI history (*β* = 0.11, 95% CI: 0.15–4.21), other comorbid chronic disease (*β* = 0.13, 95% CI: 0.41–3.91), clinical symptom (*β*  = 0.23, 95% CI: 0.23–0.61) and cardiac rehabilitation score (*β* = −0.43, 95% CI: −0.24 to 0.15) were more significantly predicted TSK and explained 46.5% of the total variance (adjusted *R*^2^ = 0.46) in CAD patients. Collinearity diagnosis showed that the variance inflation factor (VIF) ranged from 1.118 to 3.608, indicating no multicollinearity.

**Table 2:** Linear regression results with TSK.

Factors	*β* (95% CI)	*t*	*P*
Gender (male)	−0.06 (−3.24, 0.94)	−1.084	0.280
Age (61 or older)	0.07 (−0.57, 2.84)	1.31	0.192
Residential Status (living alone)	0.17 (1.53, 6.45)	3.201	0.002
Residence (city)	0.16 (0.16, 5.05)	2.101	0.037
Education			
college degree and above			
Senior high school or technical secondary school	−0.07 (−3.33, 1.26)	−0.886	0.377
Junior high school and below	−0.06 (−3.94, 1.85)	−0.714	0.476
Monthly income (RMB)			
>0.8 w			
0.5–0.8 w	−0.07 (−3.50, 1.37)	−0.882	0.379
<0.5 w	−0.01 (−3.25, 2.83)	−0.135	0.893
PCI history (yes)	0.11 (0.15, 4.21)	2.113	0.036
Other comorbid chronic disease (yes)	0.13 (0.41, 3.91)	2.434	0.016
Clinical symptom score	0.23 (0.23, 0.61)	4.373	<0.001
Cardiac rehabilitation score	−0.43 (−0.24, −0.15)	−8.014	<0.001

### Qualitative findings

A total of 18 patients (7 females, 11 males) aged 32–76 years (59.5 ± 17.5) were interviewed and identified as P1 through P18. Further detailed characteristics are presented in Table 3. Fourteen open codes were collapsed into eight focused codes, which subsequently resulted in the following four themes.

**Table 3:** Baseline characteristics of participants in the semi-structured interviews.

Identifier	Gender	Age (years)	Residential Status	Residence	PCI history	Other comorbid chronic disease	Clinical symptom scores	Cardiac rehabilitation scores	TSK scores
Participant 1	M	54	Together	City	Yes	No	3	88	51
Participant 2	M	75	Together	Town	Yes	Yes	6	66	52
Participant 3	M	64	Alone	City	No	No	10	42	61
Participant 4	F	72	Alone	City	Yes	Yes	15	82	68
Participant 5	M	43	Together	Town	No	No	3	106	39
Participant 6	F	55	Together	Town	Yes	No	11	87	55
Participant 7	M	59	Alone	City	No	No	5	74	44
Participant 8	M	45	Together	City	No	No	9	84	60
Participant 9	F	68	Alone	City	Yes	Yes	4	81	52
Participant 10	M	60	Together	Town	No	Yes	14	57	65
Participant 11	F	63	Alone	City	No	No	12	78	61
Participant 12	M	58	Together	City	Yes	No	7	100	46
Participant 13	F	76	Together	Town	No	Yes	0	99	38
Participant 14	M	60	Together	City	Yes	No	7	93	48
Participant 15	F	55	Together	Town	Yes	No	11	78	62
Participant 16	M	64	Alone	City	No	No	10	44	67
Participant 17	F	68	Alone	City	Yes	Yes	4	88	56
Participant 18	M	32	Alone	City	No	No	14	92	60

#### Theme 1: catastrophic interpretation of somatic sensations

Patients described a pattern of misinterpreting benign or normal post-operative physical sensations as signs of imminent medical danger, directly triggering fear.

#### Representative narratives

“I've had no energy since the surgery. If I walk just a little bit faster, I get out of breath. I never know if it's my heart or my lungs acting up. It makes me feel like my body has become incredibly fragile and just can't handle any kind of strain anymore.” (P4)

“This lingering fatigue and shortness of breath must mean my heart is permanently damaged and too fragile for any exertion.” (P8, P18)

“The tightness in my chest when I walk upstairs feels exactly like before my heart attack. I immediately stop, convinced it's happening again.” (P10)

*Underlying meaning:* Patients formed a direct cognitive association between physical activity (e.g., climbing stairs), the subsequent onset of somatic sensations (e.g., shortness of breath, racing heart), and the catastrophic belief of recurrent cardiac events. This created a potent “activity-sensation-fear” conditioning loop, where bodily feelings serve as an internal alarm, leading to immediate activity avoidance.

#### Theme 2: knowledge deficits and misinformation sustaining fear

A prevalent lack of clear, personalized, and authoritative information on cardiac rehabilitation emerged as a fundamental barrier, fostering uncertainty and perpetuating fear.

#### Representative narratives

“What is ‘moderate' activity? How fast should I walk? In the absence of clear guidance, I restrict my physical activity to exercises that do not provoke any discomfort.” (P2, P16)

“I was told to ‘rest well and don't overexert yourself.’ But what exactly does ‘don't overexert’ mean? I have no clear idea. My family members advise me to rest more and refrain from intense outdoor activities.” (P7)

“After returning home, I gradually resumed my previous activities, including occasional slow jogging, without experiencing any discomfort. However, I believe a formal cardiac rehabilitation plan would require professional guidance from my doctor.” (P17)

*Underlying meaning:* The absence of structured patient education resulted in reliance on the “rest-is-best” paradigm and non-authoritative information sources. This led to significant knowledge gaps regarding safe exercise parameters (type, intensity, duration) and misconceptions about stent safety, leaving patients without a cognitive framework to challenge their fears.

#### Theme 3: the vicious cycle of symptom misinterpretation and knowledge gaps

The most critical finding was the synergistic, negative feedback loop between Themes 1 and 2, where knowledge gaps and symptom misinterpretation mutually reinforced each other, solidifying kinesiophobia.

#### Representative narrative

“Whereas I was once able to walk to the market, I now experience chest tightness even during brief walks within my residential compound. This has led me to question whether the procedure was fully successful, as my original symptoms seem to persist. I initially believed I could gradually increase my activity, but now I am plagued by the apprehension that a new cardiac event is imminent.” (P6, P15)

“I felt a palpitation while walking and got scared, so I stopped. Since no one told me this could be normal, I now believe my heart can't handle simple walks.” (P11, P16)

*Underlying meaning:* This theme described a self-perpetuating cycle: ① Initial Trigger: A somatic sensation occurred during or after activity. ② Faulty Appraisal: Due to knowledge deficits (Theme 2), the sensation was catastrophically interpreted as a threat (Theme 1). ③ Behavioral Response: This appraisal elicited fear and led to activity avoidance/de-conditioning. ④ Reinforcement: De-conditioning lowered the symptom threshold, making sensations more likely to recur with less exertion. The recurrence then “confirmed” the patient's initial catastrophic belief, further deepening the fear and strengthening the avoidance behavior. This cycle entrapped patients in a state of chronic kinesiophobia.

#### Theme 4: positive perception

Interviews also revealed narratives from patients with lower kinesiophobia, which reinforced the importance of the identified themes. This highlighted that clear, personalized guidance could provide a cognitive framework to correctly interpret somatic sensations, thereby breaking the vicious cycle and enabling confident, gradual re-engagement with physical activity.

#### Representative narrative

“At the beginning, I also felt short of breath when walking. But the cardiac rehab specialist explained that this was often due to deconditioning, not necessarily my heart. She designed a slow, step-by-step plan for me. Now when I feel it, I don't panic. I see it as my body getting stronger, and I know I'm within my safe limits.” (P1)

“I had a scheduled check-in with the nurse every week [that] gave me the courage to try. It felt like having a safety net. I knew that if something felt wrong, I had someone to ask immediately, and I wouldn't be alone with my worries.” (P5)

“I was given clear guidelines: to use the Borg scale and keep my heart rate below. Knowing the parameters helps me distinguish between safe exertion and a real warning sign.” (P12, P14)

### Integrated findings: A proposed explanatory framework based on the fear-avoidance model

**Figure 1:**
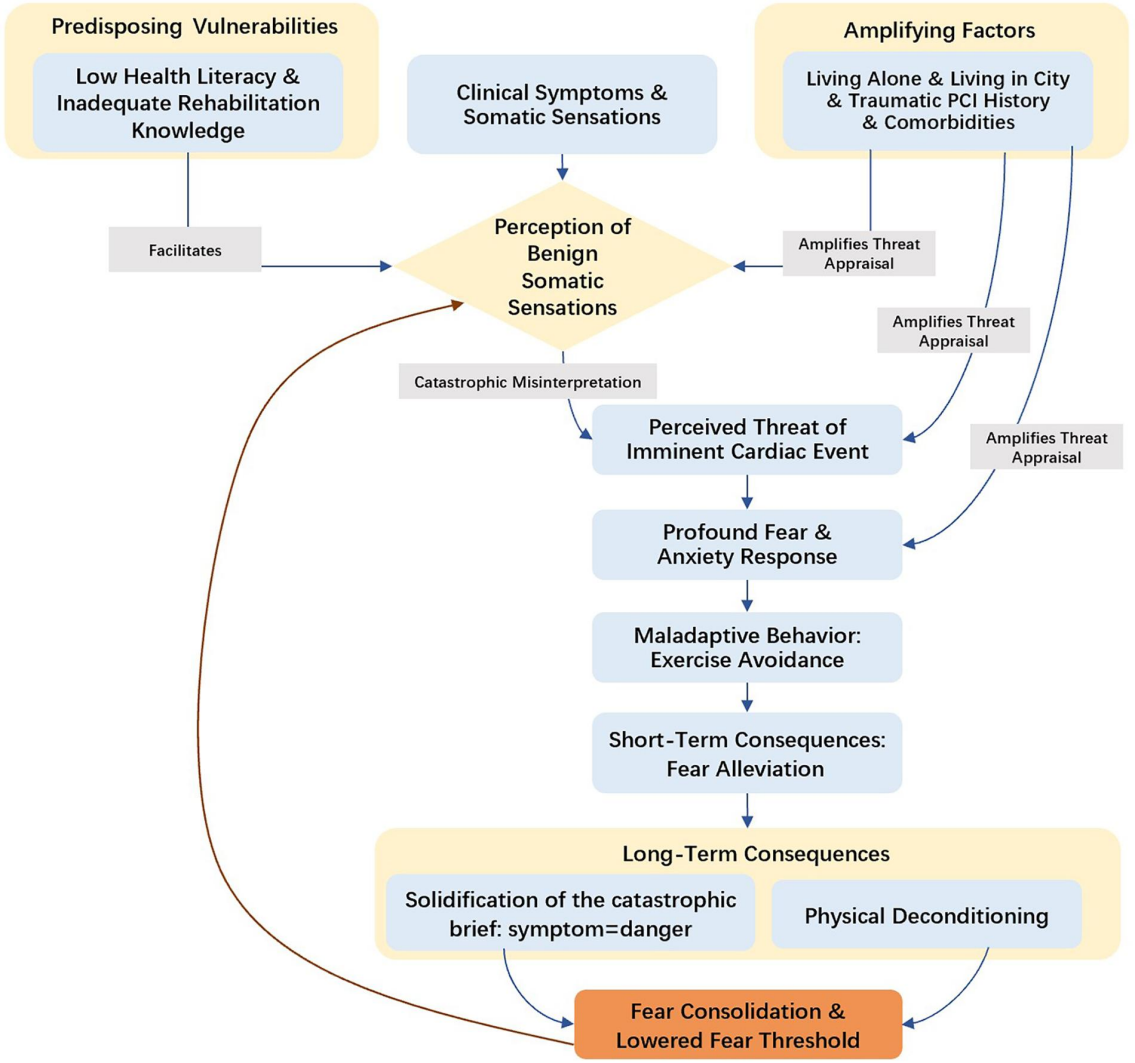
An extended fear-avoidance model of kinesiophobia in post-PCI patient.

A joint display (Table 4) was constructed to align quantitative predictors with qualitative themes. Based on this integrated analysis, we propose an explanatory framework extending the Fear-Avoidance Model by specifying the role of patient characteristics within the fear-avoidance cycle. This framework is presented as an empirically grounded interpretation derived from the integrated findings. Based on the integrated findings, we propose an explanatory framework derived from the Fear-Avoidance Model (22). This framework outlines three potential pathways underlying kinesiophobia (Figure 1).

**Table 4:** Joint display of quantitative predictors and qualitative evidence.

Quantitative Predictor	Qualitative Theme/Evidence	Integrated Interpretation
Living alone	*Theme 2*: Patients living alone reported uncertainty in managing post-discharge symptoms without immediate family support. *“My family members advise me to rest more… but no one can monitor me daily” (P7).*	Complementarity: The quantitative finding identified living alone as a significant predictor of kinesiophobia. Qualitative evidence elucidates the underlying mechanism: living alone reduces access to real-time emotional support and symptom monitoring, increasing reliance on self-directed (often overly cautious) activity restriction.
Urban residence	*Theme 2 & Positive Deviations*: Urban patients paradoxically reported both higher fear and better access to information. Some noted: “*In the absence of clear guidance, I restrict my physical activity…” (P16). “At the beginning, I also felt short of breath … specialist explained that this was often due to deconditioning” (P1).*	Complementarity *&* Expansion: The quantitative finding identified urban residence as a significant predictor of kinesiophobia. Qualitative evidence explains this seemingly paradoxical association: information abundance without personalized guidance may increase uncertainty. Positive deviance narratives further expand this understanding by revealing that when patients received structured support (e.g., scheduled check-ins, clear parameters), the “resource advantage” of urban residence became protective. This suggests that *information accessibility* alone is insufficient without* interpretive guidance.*
History of PCI	*Theme 1*: Patients with prior PCI described heightened vigilance. “*This has led me to question whether the procedure was fully successful, as my original symptoms seem to persist*” *(P6)*.	Complementarity: The quantitative finding identified prior PCI as a significant predictor of kinesiophobia. Qualitative evidence elucidates the mechanism: prior cardiac events create a learned association between physical exertion and threat, sensitizing patients to somatic cues and reinforcing catastrophic interpretations of benign post-operative sensations.
Comorbid chronic diseases	*Theme 1*: Patients with comorbidities (e.g., diabetes, COPD) expressed difficulty distinguishing symptoms. *“I never know if it's my heart or my lungs acting up” (P4).*	Complementarity: The quantitative finding identified comorbid chronic diseases as a significant predictor of kinesiophobia. Qualitative evidence reveals the underlying pathway: multiple chronic conditions complicate symptom attribution, increasing the likelihood that benign sensations are misinterpreted as cardiac threats, thereby intensifying fear.
Higher clinical symptom scores	*Theme 1*: Patients with higher symptom burden consistently reported catastrophic interpretations. *“This lingering fatigue must mean my heart is permanently damaged” (P8, P18).*	Complementarity: The quantitative finding identified higher clinical symptom burden as the strongest predictor of kinesiophobia. Qualitative evidence explains the core mechanism: greater symptom severity directly fuels catastrophic appraisal, forming the “activity-sensation-fear” conditioning loop.

Origins of Fear: The cycle may be initiated when predisposing vulnerabilities (e.g., low cardiac rehabilitation knowledge) drive catastrophic misinterpretation of benign somatic sensations, suggesting that patient characteristics may serve as key initiators.

Amplification and Escalation: Amplifying factors (e.g., living alone, urban residence, prior PCI, comorbidities) may intensify threat appraisal by heightening perceived risk and fear responses without necessarily initiating the cycle.

Behavioral Reinforcement and Consolidation: Perceived threat may lead to activity avoidance, which could prevent disconfirmation of catastrophic beliefs, potentially resulting in physical deconditioning and fear consolidation that reinforce and perpetuate the cycle.

In summary, this proposed framework illustrates how individual characteristics might function as integral components in the initiation, amplification, and perpetuation of the maladaptive fear-avoidance cycle.

## Discussion

Based on the integrated findings presented in the joint display, we propose an explanatory framework derived from the Fear-Avoidance Model. Quantitative findings identify a risk profile characterized by urban residence, PCI history, living alone, and comorbid chronic conditions. These elements may collectively exert influence through environmental, experiential, and psychosocial pathways. Urban environments characterized by a heightened tempo and sensory overload (e.g., traffic congestion, noise pollution) may contribute to elevated self-perceived stress levels (29). This increased autonomic stress response heightens patients' vigilance toward cardiorespiratory sensations such as tachycardia and dyspnea during physical activity (30). The integrated findings suggest that urban participants frequently engaged in catastrophic interpretations of benign symptoms—exemplified by equating exertional dyspnea with pre-infarction states. The absence of in-home companionship or emotional support may exacerbate exercise-related anxiety in individuals living alone (31, 32). Interview findings further indicate that socially isolated patients exhibited elevated fear levels attributable to inadequate support systems. A history of PCI may constitute psychological trauma that engenders deeply ingrained disease-specific fear memories, particularly in patients with acute myocardial infarction (33). The participant statement “It feels identical to my heart attack” epitomizes such trauma reactivation. Concurrently, comorbid conditions (e.g., diabetes mellitus, hypertension) may amplify kinesiophobia through increased baseline symptom burden (e.g., persistent fatigue) and diagnostic ambiguity (34).

Quantitative findings reveal a strong correlation between clinical symptom scores and the level of kinesiophobia, further supported by high explanatory power in regression analyses. This suggests that pronounced somatic sensations during exercise may amplify fear (15). Notably, the reappearance of symptoms similar to those preceding a past attack can trigger classical conditioning (35), potentially causing a patient's “fear of the symptoms” to thereby manifest as a fear of movement (36). Studies have shown that Post-Traumatic Stress Disorder (PTSD) is prevalent yet often goes undiagnosed in patients with coronary heart disease (37, 38). As a result, the true reason for exercise cessation may be concealed. Our qualitative data reveal that patients consistently engage in what cognitive psychology terms “catastrophic misinterpretation” (39), which refers to the tendency of attributing benign bodily sensations to imminent pathological threats. This process exemplifies the “nocebo effect” in exercise rehabilitation, where negative expectations directly shape physiological and behavioral outcomes (40). Patients' descriptions of “chest tightness identical to pre-heart attack sensations” illustrate how interoceptive awareness may become conditioned to fear responses. The immediate behavioral consequence is an overprotective avoidance pattern that follows the classical “fear-avoidance” models (41). It is noteworthy that the misinterpretation pattern persists independently of objective cardiac risk. Patients often avoid EBCR due to sensations of a racing heart or increased respiratory rate, although these are normal somatic experiences during the cardiac recovery phase. The divergence between an individual's subjective experience and objective medical reality underscores the critical importance of subjective cognitive assessment.

Furthermore, Insufficient knowledge of cardiac rehabilitation may impair patients' ability to accurately interpret bodily signals (42). Quantitative findings confirm a strong association between the level of CR knowledge and kinesiophobia, partially explaining the underlying causes. Subsequently, qualitative data highlight that incomplete exercise prescriptions, namely those lacking clear guidance on duration, intensity, and termination criteria, may lead to exercise avoidance or unguided rehabilitation, which prevents the establishment of a scientifically grounded rehabilitation regimen. This insufficient or biased rehabilitation knowledge may lead to a greater reliance on subjective reasoning over evidence-based scientific judgment (43). Research indicates that higher levels of personal mastery and stronger beliefs in one's control over life and illness are associated with greater motivation for health-promoting behaviors, more active and positive thinking patterns, and a greater tendency to adopt proactive coping strategies (44). Consequently, CAD patients may experience an erosion of these control beliefs specifically regarding disease and exercise management due to their unfamiliarity with and inability to master cardiac rehabilitation, which in turn generates significant kinesiophobia.

## Conclusion

In summary, the integrated findings suggest a self-perpetuating, negative feedback loop that perpetuates kinesiophobia. This cycle is often initiated by the catastrophic misinterpretation of benign bodily sensations in patients with a traumatic PCI history (45). Within a context of low health literacy and inadequate rehabilitation knowledge, these symptoms are not perceived as normal but as imminent threats of a recurrent cardiac event. In the absence of corrective information, this perceived threat triggers a profound fear response that is behaviorally reinforced through avoidance, thereby leading the patient to cease physical activity to achieve immediate fear reduction. However, this avoidance has the paradoxical effect of preventing disconfirmation of the catastrophic belief. The patient never learns that the symptom would have subsided without harm. Consequently, the fear is solidified, lowering the threshold for future fear responses and establishing a maladaptive pattern where any subsequent bodily sensation is more likely to be misinterpreted, thereby re-initiating the cycle with greater intensity. Factors such as living alone and comorbidities further amplify the perceived threat at each stage, accelerating this pathogenic loop.

### Strengths and limitations

This study has several notable strengths. Several limitations warrant consideration. First, the proposed explanatory framework is derived from a cross-sectional quantitative design and qualitative interviews, limiting causal inference. Although purposive sampling ensured inclusion of information-rich cases for qualitative inquiry, the limited sample size may affect generalizability. Second, while multiple regression explained 46.5% of variance in kinesiophobia scores, unmeasured psychological and environmental factors likely contribute to the phenomenon. Finally, the single-country setting necessitates caution when extrapolating findings to other healthcare systems and cultural contexts. Future research would benefit from longitudinal designs to track temporal dynamics of kinesiophobia, incorporation of objective physical activity monitoring, and development of culturally adapted interventions targeting the identified pathways.

## Data Availability

The original contributions presented in the study are included in the article/Supplementary Material, further inquiries can be directed to the corresponding author.
